# Glucose intolerance and gestational diabetes risk in relation to sleep duration and snoring during pregnancy: a pilot study

**DOI:** 10.1186/1472-6874-10-17

**Published:** 2010-05-14

**Authors:** Chunfang Qiu, Daniel Enquobahrie, Ihunnaya O Frederick, Dejene Abetew, Michelle A Williams

**Affiliations:** 1 Center for Perinatal Studies, Swedish Medical Center, Seattle, Washington, USA; 2 Department of Epidemiology, School of Public Health, University of Washington, Seattle, Washington, USA

## Abstract

**Background:**

Insufficient sleep and poor sleep quality, considered endemic in modern society, are associated with obesity, impaired glucose tolerance and diabetes. Little, however, is known about the consequences of insufficient sleep and poor sleep quality during pregnancy on glucose tolerance and gestational diabetes.

**Methods:**

A cohort of 1,290 women was interviewed during early pregnancy. We collected information about sleep duration and snoring during early pregnancy. Results from screening and diagnostic testing for gestational diabetes mellitus (GDM) were abstracted from medical records. Generalized linear models were fitted to derive relative risk (RR) and 95% confidence intervals (95% CIs) of GDM associated with sleep duration and snoring, respectively.

**Results:**

After adjusting for maternal age and race/ethnicity, GDM risk was increased among women sleeping ≤ 4 hours compared with those sleeping 9 hours per night (RR = 5.56; 95% CI 1.31-23.69). The corresponding RR for lean women (<25 kg/m^2^) was 3.23 (95% CI 0.34-30.41) and 9.83 (95% CI 1.12-86.32) for overweight women (≥ 25 kg/m^2^). Overall, snoring was associated with a 1.86-fold increased risk of GDM (RR = 1.86; 95% CI 0.88-3.94). The risk of GDM was particularly elevated among overweight women who snored. Compared with lean women who did not snore, those who were overweight and snored had a 6.9-fold increased risk of GDM (95% CI 2.87-16.6).

**Conclusions:**

These preliminary findings suggest associations of short sleep duration and snoring with glucose intolerance and GDM. Though consistent with studies of men and non-pregnant women, larger studies that include objective measures of sleep duration, quality and apnea are needed to obtain more precise estimates of observed associations.

## Background

Insufficient sleep duration and poor sleep quality are considered to be endemic in modern society. Findings from epidemiological and animal experimental studies indicate that chronic partial sleep loss is associated with increased risks of obesity and a myriad of obesity-related disorders including impaired glucose tolerance, hypertension, diabetes, metabolic syndrome, coronary heart disease, stroke and premature mortality [1-8]. The impact of short sleep duration on the risk of diabetes has been shown in several epidemiological studies, with significant increases in the incidence of diabetes among individuals who report habitual short sleep duration and those who report having difficulty maintaining sleep [4,9-11]. Moreover, accumulating evidence link habitual snoring and sleep apnea, both part of a spectrum of sleep-related breathing disorders, with cardiometabolic abnormalities that include hypertension, dyslipidemia, obesity, hyperglycemia, insulin resistance and type 2 diabetes [2,7,12-15]. Although the exact mechanisms underlying these associations have yet to be fully elucidated, evidence from experimental studies suggest that insufficient sleep and sleep fragmentation result in metabolic and neuroendocrine alterations, particularly alterations in the hypothalamic-pituitary adrenal (HPA) axis, that may contribute to the development of impaired glucose tolerance, insulin resistance and type 2 diabetes mellitus [16-20]. Moreover, experimental studies in humans [21,22] and animals [23,24] have demonstrated that intermittent hypoxia, known to occur in sleep apnea, exerts adverse effects on glucose metabolism. Most sleep studies, however, have excluded pregnant women; hence very little is known about how insufficient sleep and sleep disordered breathing during gestation contribute to increased risks of medical complications of pregnancy including gestational diabetes mellitus (GDM).

To the best of our knowledge no one has evaluated the impact of sleep duration and snoring on maternal glucose metabolism in pregnancy. In this pilot study, we assessed associations of maternal self-reported sleep duration and snoring during early pregnancy with glucose intolerance and a diagnosis of GDM later in pregnancy. We analyzed maternal plasma glucose concentrations 1 hour after a 50-gram oral glucose challenge as part of routine mid-pregnancy screening for GDM. We hypothesized that maternal habitual short sleep duration and snoring during early pregnancy were positively associated with post load glucose concentrations later in pregnancy and with an increased risk of clinically diagnosed GDM.

## Methods

### Study population and setting

This preliminary (pilot) study is based on data collected from a cohort of women attending prenatal care clinics affiliated with Swedish Medical Center in Seattle, Washington. The cohort study was designed to evaluate the influence of maternal diet, physical activity and the other life style factors on the occurrence of preeclampsia, gestational diabetes mellitus and other adverse pregnancy outcomes. Eligible women initiated prenatal care before 20 weeks gestation, were 18 years of age or older, could speak and read English, and planned to carry the pregnancy to term and to deliver at either hospital. At 14 weeks gestation, on average, participants reported sociodemographic, behavioral, and health characteristics in a structured interview. After delivery, study personnel abstracted data from participants' hospital labor and delivery medical records and clinic records. Between December 2003 and July 2006, 1,393 (82%) of 1,685 approached women consented to participate. We sequentially excluded 12 women with early pregnancy losses, 52 who were lost to follow-up, and 23 who did not complete the interview. We also excluded 16 women with pre-gestational diabetes. Thus, 1,290 women remained for analysis. All study procedures were approved by the Institutional Review Board of Swedish Medical Center. All participants provided written informed consent.

### Description of covariates

At the time of enrollment in the study, a 45 to 60-minute structured questionnaire was administered by a trained interviewer. Information on medical and reproductive histories and sociodemographic and lifestyle characteristics including average number of hours of sleep before and during early pregnancy was collected. Maternal average nightly sleep duration during pregnancy was ascertained by asking women the following question: "Since becoming pregnant, how many hours per night do you sleep?" A similar question was asked for sleep duration before pregnancy. Responses were recorded as integers. For bivariate analyses, we classified participants into 4 sleep duration categories: ≤ 4, 5-8, 9, and ≥ 10 hours, respectively. The cut-points were based on categorizations used in prior research [4,11]. Given that pregnant women, particularly those in the first trimester, are known to require 30-45 more minutes of sleep per night than their non-pregnant counterparts [25-27], we *a priori *defined those women who reported sleeping 9 hours per night as the reference group. Sleep disordered breathing during pregnancy was assessed by asking women about the frequency of **snoring **during the index pregnancy. Specifically they were asked "Since becoming pregnant, when you are asleep, to the best of your knowledge, have you snored?" Responses were as follows: (*i*) all of the time, (*ii*) most of the time, (*iii*) some of the time, (*iv*) a little of the time, and (*v*) none of the time. From this information, we categorized participants as snoring if their reported snoring most or all of the time; all other women were classified as non-snorers. Pre-pregnancy weight and height were also based on self-reports made during the interview. Pre-pregnancy body mass index (BMI) was calculated as weight in kilograms divided by height in meters squared.

In our study setting, according to the recommendations from the American Diabetes Association (ADA) [25], all pregnant women were screened at 24-28 weeks gestation using a 50 gram 1-hour oral glucose challenge test. Those patients who failed this screening test (glucose ≥ 140 mg/dl) were then followed-up within 1-2 weeks with a 100 gram, 3-hour oral glucose tolerance test (OGTT). We abstracted laboratory results from participants' 50 gram 1-hour glucose challenge test and from the diagnostic 100 gram 3-hour OGTT. Women were diagnosed with GDM if two or more of the 100 gram OGTT glucose levels exceeded the ADA criteria [28] as follows: fasting ≥ 95 mg/dl; 1-hour ≥ 180 mg/dl; 2-hour ≥ 155 mg/dl; 3-hour ≥ 140 mg/dl.

### Statistical analytical methods

We compared the frequency distribution of sociodemographic, lifestyle, behavioral and medical history characteristics of participants according to GDM diagnosis status. We assessed glucose intolerance by analyzing results of the 1-hour oral glucose screening test results. Linear regression procedures were used to estimate maternal mean 1-hour glucose concentrations while adjusting for confounding by maternal age and race/ethnicity. We fitted generalized linear models, using a log-link function, to derive relative risk (RR) and 95% confidence intervals (95% CIs) [29,30] of the associations between sleep duration and snoring variables with glucose intolerance and GDM risk. Separate models were fitted for sleep duration and snoring. We evaluated confounding due to several maternal characteristics. We selected potential confounders from a list of variables that were associated with sleep duration and snoring (from prior studies conducted among men and non-pregnant women) and that met criteria for confounding based on a review of the literature and assessment of potential causal relationships based on prior knowledge. We then controlled for potential confounders that changed multivariable RRs by more than 10% relative to the unadjusted RR [31]. On the basis of these criteria, we controlled for maternal age and race/ethnicity. None of the other variables listed in Table 1 were found to be confounders. Gestational age at enrollment or interview was not a confounder in this study. Given that this is the first study to be conducted among pregnant women, and that pre-pregnancy BMI may reasonably be considered a confounder or a covariate along the causal pathway between maternal habitual sleep behaviors and GDM, we were careful to assess the potential independent contributions of the sleep variables (from pre-pregnancy BMI). The distinction is important because adjustment for covariates along the causal pathway may spuriously attenuate estimates of association. Therefore, we constructed additional models that simultaneously adjusted for pre-pregnancy BMI. We also reported results after stratification so that we (and readers) could assess associations (even with a fair amount of statistical imprecision) without an over-reliance on model-based assumptions. In keeping with prior studies that sought to assess the extent to which adiposity modified associations between sleep disorders and abnormal carbohydrate metabolism [1,2,4,5,18], we also evaluated the joint effect of pre-pregnancy overweight status and snoring on GDM risk. For these analyses, we classified women by the joint distribution of pre-pregnancy lean or overweight status (<25 vs. ≥ 25 kg/m^2^) and snoring during early pregnancy (no vs. yes) thus resulting in the following categories: lean and non-snorer (the reference group); lean and snorer; overweight and non-snorer; and overweight and snorer. All analyses were performed using Stata 9.0 statistical software (Stata, College Station, TX). All reported p-values are two-tailed.

**Table 1:** Sociodemographic and other characteristics of the study subjects, Seattle, Washington, USA 2003-2006

		Gestational Diabetes Mellitus	
		
	Entire Study Cohort N = 1290	No N = 1222	Yes N = 68
Characteristics	n (%)	n (%)	n (%)
Maternal age at Interview (years)*	33.3 ± 4.4	33.2 ± 4.3	35.2 ± 5.0
<35 years	772 (59.8)	746 (61.0)	26 (38.2)
≥ 35 years	518 (40.2)	476 (39.0)	42 (61.8)
			
Maternal race/ethnicity*			
Non-Hispanic White	1133 (87.8)	1080 (88.4)	53 (77.9)
Other	157 (12.2)	141 (11.5)	15 (22.1)
			
Medical bill payment status			
Insurance	1239 (96.1)	1173 (96.0)	66 (97.1)
Medicaid	26 (2.0)	24 (2.0)	2 (2.9)
unknown	25 (1.9)	25 (2.0)	0 (0)
			
Nulliparous	768 (59.5)	727 (59.5)	41 (60.3)
			
≤ High school education	37 (2.9)	33 (2.7)	4 (5.9)
			
Unmarried	106 (8.2)	98 (8.0)	8 (11.8)
			
Smoked during pregnancy	67 (5.2)	65 (5.3)	2 (2.9)
			
No prenatal vitamins	32 (2.5)	30 (2.5)	2 (2.9)
			
Pre-pregnancy body mass index (kg/m^2^)*	23.5 ± 4.5	23.3 ± 4.4	26.6 ± 6.3
Lean (<18.5)	58 (4.5)	57 (4.7)	1 (1.5)
Normal (18.5-24.9)	908 (70.4)	876 (71.7)	32 (47.1)
Overweight (25-29.9)	229 (17.7)	208 (17.0)	21 (30.9)
Obese (≥30)	95 (7.4)	81 (6.6)	14 (20.4)

Mean ± standard deviation (SD).

*P-value < 0.05 from Student t test for continuous variable or from Chi-Square test for categorical variables.

## Results

Approximately 5.3% of the study cohort developed gestational diabetes mellitus (68 of 1,290). The socio-demographic characteristics of the study cohort (overall and by GDM status), are presented in Table 1. Overall, participants included in this analysis tended to be Caucasian, well-educated, and married. GDM cases were older, and heavier than women who did not develop the disorder. Cases were less likely to be of non-Hispanic White race/ethnicity than non-cases.

**Figure 1:**
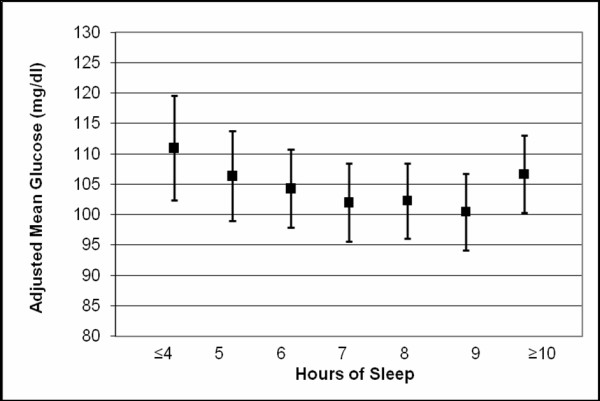
**Maternal mean plasma glucose concentrations after a 50-g glucose challenge**. Means are adjusted for maternal age and race/ethnicity. Error bars are standard errors.

**Figure 2:**
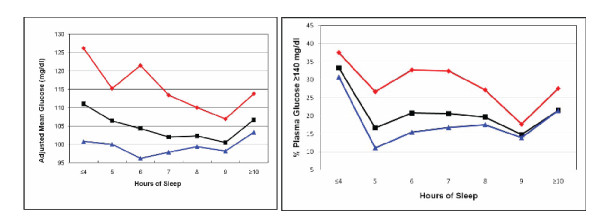
**Maternal mean 1-hour plasma glucose concentrations after a 50-g oral glucose challenge**. (a). Proportion (%) of women with 1-hour glucose concentrations ≥ 140 mg/dl (b). Values are plotted for the entire cohort (**black**), lean (body mass index < 25 kg/m^2^, **blue**) and overweight (body mass index ≥ 25 kg/m^2^, **red**) women, respectively.

A curvilinear relation was seen across levels of maternal habitual nightly sleep duration in early pregnancy for maternal mean-1 hour plasma glucose concentration after a 50-gram oral glucose challenge during weeks 24-28 gestation (Figure 1). Maternal mean 1-hour plasma glucose concentrations, adjusted for age and race/ethnicity, were highest for women who reported habitual sleep duration ≤ 4 hours per night during early pregnancy and lowest for those who reported sleeping 9 hours per night on average. Mean glucose concentrations 1-hour after a 50-gram oral glucose challenge were 16.3 mg/dl higher in women who reported sleeping ≤ 4 hours (95% CI 1.1-31.6, p = 0.04), 2.3 mg/dl higher for women who reported sleeping 5-8 hours (95% CI -1.8-6.3, p = 0.27), and 6.3 mg/dl higher for women who reported sleeping ≥ 10 hours (95% CI -0.5-13.2, p = 0.07) compared with those who reported sleeping 9 hours per night. The curvilinear relationship remained evident after participants were stratified on the basis of lean (<25 kg/m^2^) and overweight (≥25 kg/m^2^) pre-pregnancy status (Figure 2a). As can be seen in Figure 2b, the frequency distribution of women with post-50 gram 1-hour glucose concentration ≥ 140 mg/dl (i.e., the threshold for screen positive for glucose intolerance) also had a curvilinear pattern. Regardless of maternal pre-pregnancy overweight status, both short and long sleep durations were associated with higher frequencies of elevated plasma glucose concentrations (Figure 2b). Compared with women who did not snore during pregnancy, the adjusted mean 1-hour glucose concentrations were noted to be 6.4 mg/dl higher among those women who snored during pregnancy (β = 6.4 mg/dl, 95% CI 0.32-12.5, p = 0.04).

After adjusting for maternal age and race/ethnicity, women who reported sleeping ≤ 4 hours per night during early pregnancy had a 5.56-fold increased risk of GDM as compared with those women who reported sleeping 9 hours per night (the reference group) (RR = 5.56; 95% CI 1.31-23.69). The positive association remained, though was attenuated somewhat, after further adjustment for maternal pre-pregnancy BMI (RR = 4.18; 95% CI 0.94-18.60). We also noted that associations between short sleep duration and GDM risk were particularly pronounced among overweight women (far right column of Table 2). Given the suggestion of a U-shaped relation between sleep duration and GDM risk (with elevated risks for both short and long sleep duration), and given suggestion of a linear trend in risk of GDM for sleep duration ranging from ≤ 4 to 5-8 hours, we modeled the risk of GDM in relation to maternal sleep duration as a continuous variable, restricting the study population to women who reported sleeping <10 hours. In this subgroup analysis, after adjusting for maternal age and race/ethnicity, a 1-hour increase in nightly sleep was associated with a 15% reduction in GDM risk (RR = 0.85; 95% CI 0.69-1.05); though this association was not statistically significant. When analyses were restricted to overweight women, we noted that the GDM risk was reduced by 24% for each sleep hour increment (RR = 0.76; 95% CI 0.57-1.00 adjusted for maternal age and race/ethnicity), but again the association was not statistically significant. Reported sleep duration before pregnancy was highly correlated with sleep duration during pregnancy (ρ = 0.92, p < 0.002). Results were similar when analyses were repeated using maternal nightly sleep duration prior to pregnancy (data not shown).

**Table 2:** Adjusted risk ratio (RR) and 95% confidence intervals (CI) of gestational diabetes mellitus (GDM) according to self-reported sleep duration and snoring during early pregnancy.

	Gestational Diabetes Mellitus		*All	*Lean	*Overweight
				
	No N = 1222 (n)	Yes N = 68 (n)	^**1**^**Adjusted RR (95% CI)**	^***1***^***Adjusted RR (95% CI)***	^***1***^***Adjusted RR (95% CI)***
Sleep duration during pregnancy					
≤4 hours	18	3	5.56 (1.31-23.69)	*3.23 (0.34-30.41)*	*9.83 (1.12-86.32)*
5-8 hours	818	52	1.99 (0.89-4.47)	*1.28 (0.47-3.43)*	*3.47 (0.79-15.22)*
9 hours	250	7	1.00--Reference	*1.00--Reference*	*1.00--Reference*
≥10 hours	129	6	1.82 (0.60-5.57)	*1.39 (0.32-5.98)*	*2.56 (0.40-16.43)*
Missing	7	0			
					
Snore during pregnancy					
No	1112	57	1.00--Reference	*1.00--Reference*	*1.00--Reference*
Yes	80	9	1.86 (0.88-3.94)	*Insufficient data*	*2.54 (0.95-5.73)*
Missing	30	2			

*All refers to analysis completed on the entire cohort, and after restriction to lean (<25 kg/m^2^) and overweight (≥25 kg/m^2^) women, respectively.

^1^RRs and 95% CIs are adjusted for maternal age and race/ethnicity.

As can be seen from the bottom panel of Table 2, maternal snoring was associated with a statistically non-significant 1.86-fold increased risk of GDM (RR = 1.86; 95% CI 0.88-3.94). An elevated risk remained after adjustment for pre-pregnancy BMI (RR = 1.54; 95% CI 0.71-3.35). The risk of GDM was particularly elevated among overweight women who also reported snoring during pregnancy. Compared with lean women non-snorers, those women who were both overweight and who snored had a statistically significant 6.91-fold increased risk of GDM (RR = 6.91; 95% CI 2.87-16.6) (Table 3).

**Table 3:** Adjusted risk ratio (RR) and 95% confidence intervals (CI) of gestational diabetes mellitus (GDM) according to self-reporting snoring during early and pre-pregnancy overweight status.

	Gestational Diabetes Mellitus			
			
	No N = 1222 (n)	Yes N = 68 (n)	Unadjusted RR (95% CI)	^**1**^**Adjusted RR** (95% CI)
Overweight & Snore Status				
Lean & Non-Snorer	862	31	1.00--Reference	1.00--Reference
Lean & Snorer	51	1	0.55 (0.07-4.07)*	0.43 (0.06-3.28)*
Overweight & Non-Snorer	250	26	2.89 (1.69-4.96)	2.85 (1.65-4.92)
Overweight & Snorer	29	8	7.67 (3.24-18.1)	6.91 (2.87-16.6)
Missing	30	2		
	P-value for interaction		0.16	0.13

^1^RRs and 95% CIs are adjusted for maternal age and race/ethnicity

*The data are insufficient to adequately interpret relative risk estimates for lean snorers

## Discussion

Using sleep duration and snoring information, provided in early pregnancy, we were able to detect associations between sleep characteristics and maternal plasma 1-hour glucose concentrations after a 50-gram oral glucose challenge screening test later in pregnancy. Curvilinear relations were observed across nocturnal sleep duration categories. Additionally, glucose concentrations were statistically significantly elevated among women who snored during pregnancy; and the relative risk of GDM among overweight women who snored, compared to their non-snoring and lean counterparts was 6.91 (95% CI 2.87-16.6). Collectively, the findings from this pilot study provides evidence consistent with the notion that glucose homeostasis in pregnancy is sensitive to maternal habitual short sleep duration and snoring during pregnancy. To our knowledge, this is the first examination of the relation between plasma glucose concentrations, GDM risk and sleep parameters (i.e., snoring and habitual sleep duration) during pregnancy.

A large literature primarily focused on men and non-pregnant women suggest that sleep loss adversely affects glucose metabolism and increases the risk of type 2 diabetes [4,11,16,32-35]. In their cross sectional study of 740 Canadians (323 men and 417 women), Chaput et al noted that short sleep duration (<7 hours) was associated with prevalent type 2 diabetes (OR = 1.58; 95% CI 1.13-2.31) [33]. Ayas et al, in their study of 70,026 US nurses, followed for 10 years, reported that individuals who slept ≤ 5 hours per night had a significantly higher risk of symptomatic incident diabetes (OR = 1.34; 95% CI 1.04-1.72) [4]. Analysis of data from the Massachusetts Male Aging Study revealed that short sleep duration at baseline (≤5 or 6 hours per night) was associated with elevated risk of developing incident type 2 diabetes after adjustment for covariates, including age, hypertension, smoking, self-rated health, waist circumference, education, total testosterone and cortisol [11]. Further, Gangwisch et al, in their analysis of data from the first National Health and Nutrition Examination Survey (NHANES I) noted that individuals reporting ≤ 5 hours of sleep (OR = 1.47; 95% CI 1.03-2.09) and those reporting sleeping ≥ 9 hours (OR = 1.52; 95% CI 1.06-2.18) had increased risks of developing diabetes as compared to those reporting 7 hours of sleep [34]. Our findings of elevated risks of incident GDM among women reporting both long and short sleep durations in early pregnancy are consistent with these earlier reports from men and non pregnant women.

Our observations of impaired post-load glucose tolerance and increased risk of GDM among women who snore during pregnancy are also consistent with a growing body of epidemiological evidence that documents markers of the severity of obstructive sleep apnea (OSA) and increased risk of type 2 diabetes in diverse populations of men and non-pregnant women from various geographic regions [32,36-38]. In a cross-sectional study of elderly Danish men and women, investigators noted that self-reported snoring was associated with abnormal glucose tolerance test results after control for confounding factors [36]. These findings were subsequently corroborated and extended by others, including, Al-Delaimy et al [32] who reported that self-reported regular snoring was independently associated with a 2-fold increased risk (RR = 2.03; 95% CI 1.71-2.40) of developing type 2 diabetes over a 10-year follow-up period. Lindberg et al [37], in their cross sectional study of 6,799 Swedish women, for instance, reported that self-reported snoring and excessive daytime sleepiness was an independent risk factor for type 2 diabetes (OR = 1.82; 95% CI 0.97-3.43). In a population-based Swedish study of 2,668 men followed for 10-years, investigators reported that the multivariable adjusted relative risk for incident type 2 diabetes was highest for obese snorers (OR = 7.0; 95% CI 2.9-16.9) than for lean non-snorers [38]. In our study we found that the adjusted relative risk of GDM was highest among overweight mothers who snored during pregnancy (OR = 6.91; 95% CI 2.87-16.3) when compared with lean mothers who did not snore. In summary, available data support the notion that habitual snoring may be a risk factor for abnormal glucose metabolism. Our finding extends this literature by adding preliminary evidence that links snoring with pregnancy-related impaired glucose tolerance and GDM.

Our present pilot study has several important strengths. First, our determination of maternal sleep duration and snoring was based on reports made early during pregnancy, so reporting was not conditional on pregnancy outcomes or on signs and symptoms of GDM. Our results suggest that habitual short/long sleep duration and snoring precede the clinical diagnosis of GDM. Second, the high follow-up rate (>95%) minimized possible selection bias. However, several limitations merit discussion and consideration. Maternal habitual sleep duration and snoring was obtained from self-report, and thus are likely susceptible to misclassification. Reported sleep duration is known to be only moderately correlated with wrist actigraph-measured sleep duration (ρ = 0.47), and reports are generally longer by approximately 34 minutes for each hour of objectively measured sleep [39]. The use of self-reported snoring as a tool to detect sleep disordered breathing is well established. Investigators have shown that self-reported snoring correlates well with objective findings from nocturnal polysomnography, especially in frequent snorers [40]. Snoring that is infrequent or non-habitual has not been shown to be a useful screen for sleep disordered breathing in large epidemiologic studies [41,42]. It was therefore necessary to distinguish frequent snorers from infrequent snorers in our study. Our pilot study was also limited by the relatively small sample of GDM cases (n = 68) and the imprecision of relative risk estimates which were reflected by their wide 95% confidence intervals. A total of 251 study subjects had a post-50 gram 1-hour glucose concentration ≥ 140 mg/dl and thus required the follow-up diagnostic 100-gram oral glucose tolerance test (OGTT). Although qualitative results (i.e., normal/abnormal blood glucose concentrations) for the diagnostic test were available for all 251 subjects, specific glucose concentrations for each time point (i.e., fasting; 1-hour; 2-hour; and 3-hour post glucose load) were unavailable for approximately 44% of subjects. Lastly, the generalizability of our study may be limited, as our cohort was primarily comprised of Non-Hispanic White and well-educated women.

The pathophysiological mechanisms underlying these consistently observed associations of short sleep duration, sleep-related breathing disorders, including snoring, with altered glucose metabolism and diabetes are likely to be multifactorial [2,43,44]. Notably, high sympathetic nervous system activity, intermittent hypoxemia, dysregulation of the HPA axis, endothelial dysfunction and alterations in cytokine and adipokine synthesis and release have all been proposed mechanisms for these consistently observed epidemiological observations. Sympathetic hyperactivity can alter glucose homeostasis and induce insulin resistance by increasing glycogen breakdown and gluconeogenesis. Recently, investigators reported that mild sleep restriction induces marked reduction in basal glucagon concentrations [19]. Furthermore, individuals with sleep disorders may be predisposed to insulin resistance and glucose intolerance due to sleep-related dysregulation of the HPA axis with consequent elevations in serum cortisol [16,45]. In a community dwelling sample of 2,751 middle aged men and women, Kumari et al reported that short sleep duration and increased sleep disturbance are independently associated with diurnal slope in cortisol secretion [45].

Alternatively, cyclical hypoxemia with re-oxygenation, similar to repeated ischemia-reperfusion damage, may promote the formation of reactive oxygen species that may then elicit the release of pro-inflammatory cytokines including interleukin-6 and tumor necrosis factor-α [46,47]. Investigators have shown that both acute total and short-term partial sleep loss results in elevated C-reactive protein (CRP) concentrations [48]. Notably, investigators have reported that short sleep duration and other parameters of sleep disturbance during mid and late pregnancy are associated with increased systematic inflammation and higher stimulated levels of IL-6 [49,50]; and we have previously reported that early pregnancy CRP concentrations are predictive of incident GDM [51]. Collectively, these data suggest that sleep disturbances may augment pro-inflammatory responses that may then contribute to altered glucose metabolism. Causal biological mechanisms for the observed positive, though statistically in-significant association between long sleep duration and GDM are not clear. Some investigators, documenting similar positive association between long sleep duration and type 2 diabetes mellitus speculate that the association may be due in part to residual confounding by undiagnosed health conditions, co-morbid depression, unemployment, or poor general health [1,4]. Despite unclear mechanisms, the positive relationship between later pregnancy hyperglycemia and GDM risk were evident among women who reported short/long sleep duration and snoring in our cohort. The risks were particularly elevated among overweight women with these sleep disorders.

## Conclusion

These preliminary findings suggest associations of short sleep duration and snoring with glucose intolerance and GDM. Though consistent with studies of men and non-pregnant women, larger prospective studies that include objective measures of sleep duration, sleep quality, and sleep apnea during pregnancy are needed to confirm our findings. Enhanced knowledge of possible metabolic consequences of sleep disturbances in pregnancy will likely have important clinical implications in the prevention and treatment of impaired glucose tolerance and GDM among pregnant women.

## Competing interests

The authors declare that they have no competing interests.

## Authors' contributions

CQ and MAW had full access to all the data in the study and take responsibility for the integrity of the data, the accuracy of the data analysis, and the decision to submit for publication. MAW conceived, designed and obtained funding for the study. CQ analyzed the data. CQ and MAW drafted the manuscript. All authors interpreted the data, critically revised the draft for important intellectual content, and gave final approval of the manuscript to be published.

## Pre-publication history

The pre-publication history for this paper can be accessed here:

http://www.biomedcentral.com/1472-6874/10/17/prepub

## References

[B1] IOM Institute of Medicine, Committee on Sleep Medicine and Research. Sleep Disorders and Sleep Deprivation: An Unmet Public Health Problem 2006 Washington, DC: National Academy of Sciences Press 20669438

[B2] Tasali E Mokhlesi B Van Cauter E Obstructive sleep apnea and type 2 diabetes: interacting epidemics Chest 2008 133 2 496 506 10.1378/chest.07-0828 18252916

[B3] Patel SR Ayas NT Malhotra MR White DP Schernhammer ES Speizer FE Stampfer MJ Hu FB A prospective study of sleep duration and mortality risk in women Sleep 2004 27 3 440 444 15164896 10.1093/sleep/27.3.440

[B4] Ayas NT White DP Al-Delaimy WK Manson JE Stampfer MJ Speizer FE Patel S Hu FB A prospective study of self-reported sleep duration and incident diabetes in women Diabetes Care 2003 26 2 380 384 10.2337/diacare.26.2.380 12547866

[B5] Ayas NT White DP Manson JE Stampfer MJ Speizer FE Malhotra A Hu FB A prospective study of sleep duration and coronary heart disease in women Arch Intern Med 2003 163 2 205 209 10.1001/archinte.163.2.205 12546611

[B6] Resnick HE Redline S Shahar E Gilpin A Newman A Walter R Ewy GA Howard BV Punjabi NM Diabetes and sleep disturbances: findings from the Sleep Heart Health Study Diabetes Care 2003 26 3 702 709 10.2337/diacare.26.3.702 12610025

[B7] Parish JM Adam T Facchiano L Relationship of metabolic syndrome and obstructive sleep apnea J Clin Sleep Med 2007 3 5 467 472 17803009 PMC1978322

[B8] Schuster SR Tabba M Sahota P Relationship between the cardiometabolic syndrome and obstructive sleep apnea J Cardiometab Syndr 2006 1 3 204 208 10.1111/j.1559-4564.2006.05846.x 17679802

[B9] Meisinger C Heier M Loewel H Sleep disturbance as a predictor of type 2 diabetes mellitus in men and women from the general population Diabetologia 2005 48 2 235 241 10.1007/s00125-004-1634-x 15645205

[B10] Mallon L Broman JE Hetta J High incidence of diabetes in men with sleep complaints or short sleep duration: a 12-year follow-up study of a middle-aged population Diabetes Care 2005 28 11 2762 2767 10.2337/diacare.28.11.2762 16249553

[B11] Yaggi HK Araujo AB McKinlay JB Sleep duration as a risk factor for the development of type 2 diabetes Diabetes Care 2006 29 3 657 661 10.2337/diacare.29.03.06.dc05-0879 16505522

[B12] Okcay A Somers VK Caples SM Obstructive sleep apnea and hypertension J Clin Hypertens (Greenwich) 2008 10 7 549 555 10.1111/j.1751-7176.2008.07811.x 18607146 PMC8109964

[B13] Ip MS Lam B Ng MM Lam WK Tsang KW Lam KS Obstructive sleep apnea is independently associated with insulin resistance Am J Respir Crit Care Med 2002 165 5 670 676 11874812 10.1164/ajrccm.165.5.2103001

[B14] Levy P Bonsignore MR Eckel J Sleep, sleep-disordered breathing and metabolic consequences Eur Respir J 2009 34 1 243 260 10.1183/09031936.00166808 19567607

[B15] Pack AI Gislason T Obstructive sleep apnea and cardiovascular disease: a perspective and future directions Prog Cardiovasc Dis 2009 51 5 434 451 10.1016/j.pcad.2009.01.002 19249449

[B16] Spiegel K Leproult R Van Cauter E Impact of sleep debt on metabolic and endocrine function Lancet 1999 354 9188 1435 1439 10.1016/S0140-6736(99)01376-8 10543671

[B17] Spiegel K Leproult R L'Hermite-Baleriaux M Copinschi G Penev PD Van Cauter E Leptin levels are dependent on sleep duration: relationships with sympathovagal balance, carbohydrate regulation, cortisol, and thyrotropin J Clin Endocrinol Metab 2004 89 11 5762 5771 10.1210/jc.2004-1003 15531540

[B18] Spiegel K Knutson K Leproult R Tasali E Van Cauter E Sleep loss: a novel risk factor for insulin resistance and Type 2 diabetes J Appl Physiol 2005 99 5 2008 2019 10.1152/japplphysiol.00660.2005 16227462

[B19] Schmid SM Jauch-Chara K Hallschmid M Schultes B Mild sleep restriction acutely reduces plasma glucagon levels in healthy men J Clin Endocrinol Metab 2009 94 12 5169 5173 10.1210/jc.2009-0969 19837925

[B20] Schmid SM Hallschmid M Jauch-Chara K Bandorf N Born J Schultes B Sleep loss alters basal metabolic hormone secretion and modulates the dynamic counterregulatory response to hypoglycemia J Clin Endocrinol Metab 2007 92 8 3044 3051 10.1210/jc.2006-2788 17519315

[B21] Oltmanns KM Gehring H Rudolf S Schultes B Rook S Schweiger U Born J Fehm HL Peters A Hypoxia causes glucose intolerance in humans Am J Respir Crit Care Med 2004 169 11 1231 1237 10.1164/rccm.200308-1200OC 15044204

[B22] Cutler MJ Swift NM Keller DM Wasmund WL Smith ML Hypoxia-mediated prolonged elevation of sympathetic nerve activity after periods of intermittent hypoxic apnea J Appl Physiol 2004 96 2 754 761 10.1152/japplphysiol.00506.2003 14555683

[B23] Iiyori N Alonso LC Li J Sanders MH Garcia-Ocana A O'Doherty RM Polotsky VY O'Donnell CP Intermittent hypoxia causes insulin resistance in lean mice independent of autonomic activity Am J Respir Crit Care Med 2007 175 8 851 857 10.1164/rccm.200610-1527OC 17272786 PMC1899294

[B24] Polotsky VY Li J Punjabi NM Rubin AE Smith PL Schwartz AR O'Donnell CP Intermittent hypoxia increases insulin resistance in genetically obese mice J Physiol 2003 552 Pt 1 253 264 10.1113/jphysiol.2003.048173 12878760 PMC2343324

[B25] International Classification of Sleep Disorders Revised: Diagnostic and Coding Manual 2000 Rochester, MN: American Academy of Sleep Medicine 14 17

[B26] Hedman C Pohjasvaara T Tolonen U Suhonen-Malm AS Myllylä VV Effects of pregnancy on mothers' sleep Sleep Med 2002 3 1 37 42 10.1016/S1389-9457(01)00130-7 14592252

[B27] Pien GW Schwab RJ Sleep disorders during pregnancy Sleep 2004 27 7 1405 1417 15586794 10.1093/sleep/27.7.1405

[B28] ADA Gestational diabetes mellitus Diabetes Care 2004 27 Suppl 1 88 90 10.2337/diacare.27.2007.s88 14693936

[B29] Hardin JW Hilbe J Generalized Linear Models and Extensions 2001 College Station, TX: Stata Press

[B30] Baker RJ Nelder JA *The Generalized Linear Interactive Modeling System *release 3.77 1985 Oxford: Oxford Numerical Algorithms Group

[B31] Rothman KJ Greenland S Lash TL Modern Epidemiology, 3rd version 2008 Philadelphia: Lippincott Williams & Wilkins 261 263

[B32] Al-Delaimy WK Manson JE Willett WC Stampfer MJ Hu FB Snoring as a risk factor for type II diabetes mellitus: a prospective study Am J Epidemiol 2002 155 5 387 393 10.1093/aje/155.5.387 11867347

[B33] Chaput JP Despres JP Bouchard C Tremblay A Association of sleep duration with type 2 diabetes and impaired glucose tolerance Diabetologia 2007 50 11 2298 2304 10.1007/s00125-007-0786-x 17717644

[B34] Gangwisch JE Heymsfield SB Boden-Albala B Buijs RM Kreier F Pickering TG Rundle AG Zammit GK Malaspina D Sleep duration as a risk factor for diabetes incidence in a large U.S. sample Sleep 2007 30 12 1667 1673 18246976 10.1093/sleep/30.12.1667 PMC2276127

[B35] Knutson KL Van Cauter E Associations between sleep loss and increased risk of obesity and diabetes Ann N Y Acad Sci 2008 1129 287 304 10.1196/annals.1417.033 18591489 PMC4394987

[B36] Jennum P Sjol A Snoring, sleep apnoea and cardiovascular risk factors: the MONICA II Study Int J Epidemiol 1993 22 3 439 444 10.1093/ije/22.3.439 8359959

[B37] Lindberg E Berne C Franklin KA Svensson M Janson C Snoring and daytime sleepiness as risk factors for hypertension and diabetes in women--a population-based study Respir Med 2007 101 6 1283 1290 10.1016/j.rmed.2006.10.015 17127049

[B38] Elmasry A Lindberg E Berne C Janson C Gislason T Awad Tageldin M Boman G Sleep-disordered breathing and glucose metabolism in hypertensive men: a population-based study J Intern Med 2001 249 2 153 161 10.1046/j.1365-2796.2001.00787.x 11240844

[B39] Lauderdale DS Knutson KL Yan LL Liu K Rathouz PJ Self-reported and measured sleep duration: how similar are they? Epidemiology 2008 19 6 838 845 10.1097/EDE.0b013e318187a7b0 18854708 PMC2785092

[B40] Bliwise DL Nekich JC Dement WC Relative validity of self-reported snoring as a symptom of sleep apnea in a sleep clinic population Chest 1991 99 3 600 608 10.1378/chest.99.3.600 1995215

[B41] Gislason T Benediktsdóttir B Björnsson JK Kjartansson G Kjeld M Kristbjarnarson H Snoring, hypertension, and the sleep apnea syndrome. An epidemiologic survey of middle-aged women Chest 1993 103 4 1147 1151 10.1378/chest.103.4.1147 8131455

[B42] Jennum P Hein HO Suadicani P Gyntelberg F Risk of ischemic heart disease in self-reported snorers. A prospective study of 2,937 men aged 54 to 74 years: the Copenhagen Male Study Chest 1995 108 1 138 142 10.1378/chest.108.1.138 7606948

[B43] Arnaud C Dematteis M Pepin JL Baguet JP Levy P Obstructive sleep apnea, immuno-inflammation, and atherosclerosis Semin Immunopathol 2009 31 1 113 125 10.1007/s00281-009-0148-5 19404644 PMC3937574

[B44] Arnardottir ES Mackiewicz M Gislason T Teff KL Pack AL Molecular signatures of obstructive sleep apnea in adults: a review and perspective Sleep 2009 32 4 447 470 19413140 10.1093/sleep/32.4.447 PMC2663860

[B45] Kumari M Badrick E Ferrie J Perski A Marmot M Chandola T Self-reported sleep duration and sleep disturbance are independently associated with cortisol secretion in the Whitehall II study J Clin Endocrinol Metab 2009 94 12 4801 4809 10.1210/jc.2009-0555 19850688 PMC2795654

[B46] Vgontzas AN Bixler EO Chrousos GP Metabolic disturbances in obesity versus sleep apnea: the importance of visceral obesity and insulin resistance J Intern Med 2003 254 1 32 44 10.1046/j.1365-2796.2003.01177.x 12823641

[B47] Schulz R Mahmoudi S Hattar K Sibelius U Olschewski H Mayer K Seeger W Grimminger F Enhanced release of superoxide from polymorphonuclear neutrophils in obstructive sleep apnea. Impact of continuous positive airway pressure therapy Am J Respir Crit Care Med 2000 162 2 Pt 1 566 570 10934088 10.1164/ajrccm.162.2.9908091

[B48] Meier-Ewert HK Ridker PM Rifai N Regan MM Price NJ Dinges DF Mullington JM Effect of sleep loss on C-reactive protein, an inflammatory marker of cardiovascular risk J Am Coll Cardiol 2004 43 4 678 683 10.1016/j.jacc.2003.07.050 14975482

[B49] Okun ML Hall M Coussons-Read ME Sleep disturbances increase interleukin-6 production during pregnancy: implications for pregnancy complications Reprod Sci 2007 14 6 560 567 10.1177/1933719107307647 17959884

[B50] Okun ML Coussons-Read ME Sleep disruption during pregnancy: how does it influence serum cytokines? J Reprod Immunol 2007 73 2 158 165 10.1016/j.jri.2006.06.006 17074396

[B51] Qiu C Sorensen TK Luthy DA Williams MA A prospective study of maternal serum C-reactive protein (CRP) concentrations and risk of gestational diabetes mellitus Paediatr Perinat Epidemiol 2004 18 5 377 384 10.1111/j.1365-3016.2004.00578.x 15367325

